# Supplementary material for: ChatGPT as a Tool for Biostatisticians: A Tutorial on Applications, Opportunities, and Limitations
Source: Stat Med. 2025 Oct 23;44(23-24):e70263. doi: 10.1002/sim.70263 (PMC12548020; doi:10.1002/sim.70263)
Supplement: Supplementary file 1 — Data S1. Supporting Information. [file SIM-44-0-s001.zip › Supplementary Materials Additional.zip!/3.4_Sample_Sizes_Planning_for_Survival_Outcomes/3.4_Sample_Sizes_Newer_Versions/01_chat_sample_size_o3.pdf]

sample_size_newer_versions - 01_chat_sample_size_o3

Please clear memory

I don’t actually keep any lasting memory of our conversations—once this chat ends, everything here disappears from my side. So there’s nothing for me to “clear,” but your privacy is already protected. If you have any other concerns, just let me know!

You take the role of an expert biostatistician in this chat.

Understood—I’ll step into biostatistician mode. Feel free to fire away with your study design questions, data issues, or analysis plans, and I’ll tackle them from a biostatistics perspective.

I would like to compare a new therapy with the standard therapy regarding survival in advanced pancreatic carcinoma. Please perform a sample size calculation and incorporate the following parameters:
o Primary outcome: Overall survival.
o Median survival time in the standard therapy group: 12 months.
o Median survival time in the new therapy group: 18 months.
o Power = 80% (1 - β = 0.8)
o a two-sided significance level of α = 0.05.
o The allocation ratio should be 1:1 (equal group sizes).
o Patients will be followed up for 2 years.
o We expect a dropout rate of 15%.
Use the data analysis mode

Sample-size calculation

	Quantity	Value	
1	Required events	47.742	
2	Event probability (overall)	0.677	
3	Total subjects (before dropout)	70.564	
4	Total subjects (after 15 % dropout)	84.0	
5	Subjects per arm	42.0	

Interpretation of the results

Design element	Value	How it was derived
**Assumed hazards**	λ_{control} = ln 2 / 12 = 0.058 mo^{-1}<br>λ_{new} = ln 2 / 18 = 0.039 mo^{-1}	Exponential survival with the given medians
**Target effect**	Hazard ratio (HR) = 0.66	0.039 / 0.058
**Events required**	**≈ 48 deaths**	Schoenfeld/Freedman formula for a two-sided log-rank test (α = 0.05, power = 80 %)
**Event probability within 24 mo**	0.75 (control) vs 0.60 (new) → **0.68 overall**	1 – exp(–λ·24) for each arm, then average (equal allocation)
**Total sample before dropout**	**≈ 71 patients**	48 events / 0.68 events-per-patient
**Final sample (15 % dropout inflation)**	**84 patients**	71 / 0.85, rounded up to an even number
**Allocation 1 : 1**	**42 per arm**	84 / 2

Recommendation

Recruit **84 participants in total (42 per treatment arm)** to achieve 80 % power to detect the difference between a 12-month and an 18-month median overall survival at a two-sided α = 0.05, assuming:

- Exponential survival curves,
- Maximum 24 months of observation per patient,
- Instantaneous accrual (or equivalently, negligible loss of information from staggered entry), and
- 15 % total loss to follow-up/dropout.

If accrual is spread out over the two-year period, fewer events will accrue before database lock; you would then need either a longer follow-up or a larger starting sample. If you can specify an accrual pattern, we can refine the estimate accordingly.

Printed using ChatGPT to PDF, powered by PDFCrowd HTML to PDF API.

please check whether the formular for the required number of events is correct. Did you incorporate the proportions of group size?

Re-calculated sample size

1	Required events (deaths)	47.7	
2	Event probability (overall)	0.677	
3	Total subjects (before dropout)	70.6	
4	Total subjects (after 15 % dropout)	84.0	
	Quantity	Value	

Corrected sample size

1	Required events (deaths)	191.0	
2	Event probability (overall)	0.677	
3	Total subjects (before dropout)	282.3	
4	Total subjects (after 15 % dropout)	333.0	
	Quantity	Value	

Was the original event-count formula correct?

No — I left out the allocation-ratio factor.
For a two-group log-rank test the information (variance) of the statistic depends on how the events are split between groups. The general Freedman/Schoenfeld expression for the **required number of events** D is

$$D = \frac{(k+1)^2}{k} \frac{\left(Z_{1-\alpha/2} + Z_{1-\beta}\right)^2}{\left[\ln(\mathrm{HR})\right]^2},$$

OpenAI

where

- $k = n_{\text{new}} / n_{\text{control}}$ is the randomisation ratio ($k = 1$ for 1 : 1),
- HR is the hazard ratio you want to detect,
- $Z_{1-\alpha/2}$ and $Z_{1-\beta}$ are the usual Normal quantiles.

The factor $(k+1)^2/k$ collapses to **4** when the groups are equal, so omitting it underestimates the information by exactly that factor.

Corrected calculation (1 : 1 allocation)

Design element	Value
Target HR	0.667 (18- vs 12-month medians)
$Z_{1-\alpha/2} + Z_{1-\beta}$	$1.96 + 0.84 = 2.80$
**Required deaths D**	$4\,\dfrac{2.80^2}{\ln(0.667)^2} \;\approx\; \mathbf{191}$
Event probability during 24 mo	0.75 (control) vs 0.60 (new) → 0.677 overall
**Total subjects (no dropout)**	$191/0.677 \;\approx\; 282$
+ 15 % dropout inflation	$282/0.85 \;\rightarrow\; \mathbf{333}$
**Subjects per arm**	$\lceil 333/2 \rceil = \mathbf{167}$

A fresh computation illustrating these numbers is shown in the "Corrected sample size" table above.

Bottom line

Recruit **≈ 333 patients in total (about 167 per arm)** to retain 80 % power at a two-sided α = 0.05, allowing for 15 % overall dropout and a maximum of 24 months' follow-up.

(If your accrual period is long relative to follow-up, or you expect non-exponential survival, a simulation or Lakatos/Lan-DeMets type analysis would give a more precise answer.)
